# A comparative study of pathological and prognostic differences in DCIS between Asian and Caucasian women

**DOI:** 10.1186/bcr3799

**Published:** 2015-11-05

**Authors:** Raeesa Patel, Anil Jain, Philip Foden

**Affiliations:** 1 The University of Manchester, UK; 2 The Nightingale Centre and Genesis Prevention Centre, Manchester, UK

## Introduction

The aim was to compare the histopathological and prognostic differences in DCIS between age-matched Asian and Caucasian female patients.

## Methods

Data related to presentation, histopathology, prognosis and treatment of DCIS were gathered from 48 women from the Asian Breast Cancer Database at the Nightingale Centre, all of whom had begun with an initial diagnosis (at biopsy) of DCIS. These were compared with age-matched Caucasian patients, also diagnosed with DCIS at the time of biopsy. The total study included 96 patients.

## Results

Out of 48 Asian women more presented symptomatically (25, 52.1 %) compared to Caucasian women (13, 27.1 %), *p *= 0.012. Asian women had a larger mean value in regards to tumour size (28.48 mm) compared to Caucasian women (21.59 mm), and more progressed from an initial diagnosis of DCIS, to a final diagnosis of DCIS with an invasive component (12.5 % compared to 2.1 %), *p *= 0.05. However, differences in the average Van Nuys Prognostic Index score were not statistically significant in Asian (7.13) and Caucasian (7.51) patients, *p *= 0.236. Interestingly, significantly more Asian women were treated with mastectomy (47.9 %) compared to Caucasian women (22.9 %), *p *= 0.015.

## Conclusion

Asian women presented with a larger tumour size, more progressed to a diagnosis of invasive carcinoma, and more had mastectomies compared to Caucasian women. Since fewer Asian women are presenting via the screening programme, education and awareness of breast cancer and screening needs to be increased in Asian women to increase their screening uptake rates.

